# The Medical Digest, or Busy Practitioner's Vade-Mecum

**Published:** 1886-09

**Authors:** 


*The Medical Digest, or Busy Practitioner's Vade-Mecum.* Being a Means of Readily Acquiring Information upon the Principal Contributions to Medical Science during the last Thirty-five Years. Second Edition. 1882. The First Appendix to the *Medical Digest,* including the Years 1882–3–4–5 and Early Part of 1886. By RICHARD NEALE, M.D. Lond. LEDGER, SMITH and Co. 1886.

The first edition of this work, which was published by the New Sydenham Society in 1877, is not nearly so well known as it ought to be. The second edition was issued in 1882, and now Dr. Neale gives us an appendix which includes the years mentioned in the title. The work must ever remain a praiseworthy monument to the labours of one man, who thereby has laid the whole profession under a deep debt of gratitude.

Much material valuable to the practitioner lies buried in the pages of periodicals; and, although Dr. Neale's work refers to only a few of these publications, yet to get even these dealt with in this manner is a gain not easily to be estimated. We should imagine that Dr. Neale will not claim any vested interests in this kind of work; and it would, probably, be no small satisfaction to him if he knew that his arduous task had stimulated others to work at the periodicals which he has not touched.

Dr. Neale says "it has been urged that without the periodicals to which reference is made the value of the book is diminished." This objection will always have great force, because the work—although it is more than a mere bibliographical index, inasmuch as it often gives the leading thought of the paper whose title is quoted—is not a Digest in the sense of giving an epitome of a communication which might serve as a substitute for the article itself. But, nevertheless, Dr. Neale's *Digest* is indispensable to all medical authors; and every doctor in practice should be the possessor of this book, which, in such a compact form, renders so many sources of information accessible to him.

With such books as Vol. III. of the Medical and Chirurgical Society's Library-Catalogue, Dr. Billings' *Index-Catalogue*, the *Index Medicus*, Dr. Würzburg's *Medicinische Bibliographie*, and Dr. Neale's *Digest*, the man who has to get up a special subject will, in the first place, be appalled at the vast amount of literature opened out before him, and then be grateful to the workers who have, in such systematic form, given him the titles of the works that will help him.

